# Integrating indigenous knowledge and ecological modeling to understand anthrax transmission risk

**DOI:** 10.1371/journal.pntd.0014796

**Published:** 2026-11-10

**Authors:** Juan Martin Dabezies, Maria Zubkova, Christian Fohringer, Kevin M. Curtin, Delong He, Henriette van Heerden, Meredith L. Gore

**Affiliations:** 1 Department of Agrarian Systems and Cultural Landscapes, Universidad de la República, Rocha, Uruguay; 2 Department of Geographical Sciences, University of Maryland, College Park, Maryland, United States of America; 3 Department of Ecoscience, Aarhus University, Roskilde, Denmark; 4 Department of Geography and the Environment, Laboratory for Location Science, University of Alabama, Tuscaloosa, Alabama, United States of America; 5 Department of Veterinary Tropical Diseases, University of Pretoria, Pretoria, South Africa; Oklahoma State University, UNITED STATES OF AMERICA

## Abstract

Anthrax, caused by *Bacillus anthracis*, remains a persistent zoonotic threat across African savannas, yet its transmission dynamics are poorly understood in regions with limited surveillance systems. Northern Limpopo National Park (Mozambique) combines ecological conditions favorable for spore persistence with Indigenous communities that depend on unfenced grazing and frequent wildlife contact. We integrated ecological suitability modeling with participatory mapping of extensive cattle herding practices to generate fine-scale risk surfaces for anthrax transmission. Ecological modeling indicated broad suitability for spore survival, while participatory mapping revealed localized exposure risk zones. The integrated model refined these patterns by identifying areas where broad ecological suitability intersected with locally mapped livestock use and potential exposure pathways. While the highest suitability class did not consistently coincide with mapped high-exposure features, most participatory risk layers fell within moderately suitable zones, suggesting that fine-scale transmission concern is structured by everyday grazing, water use, and mobility within an environmentally favorable landscape. This integration captures pathogen persistence potential and context-specific exposure dynamics, providing a socially grounded approach to risk prioritization in the absence of robust outbreak records. The findings help inform contextualized and targeted prevention and response options—such as risk communication, vaccination campaigns, and community-based surveillance—and illustrate how combining ecological modeling with Indigenous spatial knowledge can help strengthen zoonotic risk assessments in under-surveilled settings.

## Author summary

Anthrax is a serious disease of livestock and wildlife that can also infect people. It is caused by a bacterium whose spores can remain in soil for many years. In rural areas with limited veterinary surveillance, outbreaks may go undetected even where environmental conditions favor the disease. We studied three Indigenous pastoral communities in northern Limpopo National Park, Mozambique, where cattle graze in open, unfenced landscapes under community-guided herding practices and share space with wildlife. We combined ecological modeling based on climate, soil, vegetation, elevation, and water with participatory mapping of grazing areas, water sources, livestock routes, and places where animal sickness or deaths had been observed. The ecological model showed that most community lands were suitable for anthrax spore persistence, while participatory mapping identified more localized areas of potential exposure. These two types of risk did not always overlap. Our results show that combining environmental data with local knowledge can identify where livestock are most likely to encounter risk and can help guide targeted vaccination, risk communication, and community-based surveillance where formal outbreak records are limited.

## 1 Introduction

Anthrax, caused by *Bacillus anthracis*, is a zoonotic disease of significant public health and economic concern, yet its transmission dynamics remain poorly understood in many endemic rural areas [1]. The disease primarily affects herbivores, including domestic animals such as cattle and wildlife like elephants, which become infected by ingesting spores present in soil or on contaminated vegetation. The spores of *B. anthracis* are highly resilient and can survive for decades under favorable environmental conditions [2]. In several African countries, recurring anthrax outbreaks have affected domestic animals and wildlife as well as humans, with substantial impacts on biodiversity and rural livelihoods [3–6].

In southern Africa, where the pathogen is endemic, anthrax transmission occurs through several pathways that facilitate ingestion of or contact with *B. anthracis* spores [2,7,8]. Herbivores are typically infected while grazing in contaminated pastures, where spores persist in the soil or adhere to vegetation [4,9–14]. Wildlife interactions further amplify transmission risks, as carcass-feeding animals, including vultures, blowflies, hyenas, and lions, can disseminate spores to nearby vegetation or water sources [8,15]. Direct contact with animal carcasses also represents an exposure route, as these carcasses can release large quantities of spores into the environment. Collectively, grazing, carcass contact, and use of shared water sources are key transmission pathways in the multi-host environments. Thus, the ecology of environmentally persistent pathogens such as *B. anthracis* reflects the spatially explicit convergence of environmental suitability (i.e., persistence) and host interactions (i.e., exposure) [16].

In sub-Saharan Africa, effective anthrax management remains a persistent challenge at the human-livestock-wildlife interface. The disease is endemic in conservation areas, communal rangelands, and transboundary ecosystems across countries such as Tanzania, Kenya, Namibia, Zimbabwe, and South Africa [5,15]. These regions are often characterized by dry seasons, limited veterinary infrastructure, and high livelihood dependence on traditional pastoral systems—conditions that can facilitate pathogen persistence and circulation, and complicate outbreak detection. Despite the recurrent nature of anthrax outbreaks in these settings, the lack of fine-scale surveillance data and limited integration of local knowledge continue to hinder targeted prevention strategies essential for effective disease management [17].

In the northern regions of Kruger National Park (KNP) in South Africa, for example, anthrax outbreaks in wildlife have been well documented with active surveillance since the 1980s [8,18]. The region’s alkaline soils, rich in calcium, promote *B. anthracis* spore persistence, while warm and arid climates support its survival [8]. Seasonal water bodies, which attract large concentrations of wildlife, further amplify the risk of disease transmission [16,17,19,20]. This interplay creates an environment for sporadic anthrax re-emergence, posing challenges to wildlife conservation and health management [5,21].

Mozambique’s northern Limpopo National Park (LNP) shares many ecological features that favor anthrax persistence with the adjacent northern KNP, particularly across their shared border, where outbreaks have been repeatedly documented in KNP [8]. On the Mozambican side of this border, however, no confirmed outbreaks have been reported despite the clear ecological continuity. The absence of records likely reflects limited monitoring capacity rather than absence of disease, as Mozambique has only submitted scarce anthrax outbreak data to the World Organization for Animal Health (WOAH), with only a few reports in 2017 and 2024 [The recent 2024 notification features only domestic cattle (https://wahis.woah.org/#/in-review/5588). Previous data from 2017-2018 has some more information (https://wahis.woah.org/#/in-review/2347?fromPage=event-dashboard-url).]. This contrast underscores a critical asymmetry between the two parks: KNP has long benefited from consolidated surveillance systems and veterinary infrastructure and has no resident human population, whereas LNP combines weaker surveillance capacity and limited veterinary services with resident Indigenous communities living within the park landscape [22]. These communities are reliant on traditional pastoralism and coexist with wildlife under conditions of weak veterinary services and limited disease monitoring, creating a socio-ecological interface where human, livestock, and wildlife interactions amplify transmission potential. In this context, exposure risk is likely to be present but poorly detected, making conventional surveillance-based approaches insufficient and motivating proxy methods that combine ecological suitability with locally grounded exposure information.

Identifying areas suitable for *B. anthracis* is essential for anticipating future outbreaks and guiding prevention strategies [8]. Several studies highlight the importance of developing suitability maps based on ecological drivers, leveraging Geographic Information Systems (GIS), anthrax outbreak records, and global geodatabases to address information gaps in this field [16,19,23–25]. These analyses are particularly challenging in settings where systematic outbreak records are lacking or incomplete. In such contexts, combining ecological suitability analyses with local knowledge is one mechanism for improving spatial precision and generating context-specific insights into exposure pathways [17]. Several studies show that incorporating host abundance or livestock density alongside environmental predictors improves anthrax suitability models [26,27].

Local knowledge plays a fundamental role in identifying disease transmission pathways, perceived risk areas, and patterns of interaction between livestock and wildlife [9,28–32]. Moreover, incorporating local knowledge strengthens the legitimacy and flexibility of planning processes, particularly in complex socio-ecological systems where exclusion often fosters mistrust and leads to less effective outcomes [33]. In this regard, participatory mapping has proven to be effective at measuring local insights about disease risks, enabling the identification of cattle movement patterns, livestock management practices, and hotspots of livestock–wildlife interactions that would remain unidentified by solely using empirical data or existing databases [9,10,12,13,34].

This study integrates ecological modeling with indigenous knowledge of grazing and animal keeping, representing host abundance, within a GIS framework to assess anthrax transmission risk to cattle in three Indigenous pastoral communities of northern LNP, Mozambique. In a region where anthrax remains a persistent yet under-documented threat at the wildlife–livestock–human interface, this research addresses the lack of fine-scale, community-grounded risk assessments by combining ecological variables (e.g., vegetation, elevation, water proximity, and soil properties) with participatory mapping of livestock practices and movement patterns. The study is structured around three components: [1] ecological risk assessment using technical methods; [2] assessment of risk perception using participatory methods; and [3] integration of risk assessment and perceptual data for a more holistic understanding of disease transmission risk.

## 2. Methods

### 2.1 Ethics approval and consent to participate

This study did not involve experiments with humans or their tissues. Data collection activities involving human subjects and field access were conducted under the relevant institutional and national authorizations. In the United States, the study was reviewed and approved as Exempt Human Subjects Research by the U.S. Army Medical Research and Development Command, Office of Human Research Oversight (Decision: Exempt, Mozambique-Specific Research Activities; OHRO Log Number E05089, February 5, 2024), the University of Maryland Institutional Review Board (Decision: Exempt category #45CFR46.104[d][2][ii]; Clearance number 2068333–1, July 17, 2023), and the University of Alabama Institutional Review Board (Exempt category #45CFR46; e-protocol 23-08-6834, March 18, 2024). In Mozambique, approval and authorization were granted by Limpopo National Park and the Administração Nacional das Áreas de Conservação (Decision: Approved, November 3, 2023), as well as by Universidade de Licungo (Decision: Exempt, December 19, 2023). Permissions to access communities and participants were also obtained from local authorities. Oral informed consent was obtained from all participants or their legal guardians (S1 Material). All methods were carried out in accordance with relevant guidelines and regulations. Data were analyzed anonymously, ensuring the confidentiality of participants, and community names were anonymized throughout the manuscript.

### 2.2 Study area

LNP is a 9,260 km^2^ protected area in the Gaza Province of Mozambique. It forms part of the Greater Limpopo Transfrontier Conservation Area (GLTFCA), encompassing nearly 100,000 km^2^, which includes the Kruger National Park (KNP) in South Africa, Gonarezhou National Park (GNP) in Zimbabwe, and additional areas in Mozambique. LNP is a cornerstone of the GLTFCA, functioning as a transboundary conservation area. The underexplored northern LNP, bordering KNP and GNP, is crucial for conservation and disease surveillance, as its sandy savannas and alluvial floodplains facilitate both wildlife movement and anthrax spore deposition. Like KNP’s Pafuri and Shingwedzi regions, which have been identified as anthrax hotspots in part due to high soil calcium, neutral-to-alkaline pH, seasonal water dynamics [8], and anthrax outbreaks [18], the northern LNP Pafuri area likely exhibits comparable environmental conditions.

Indigenous communities inhabiting LNP belong to the Shangaan/Tsonga ethnic group and play a pivotal role in the park’s socio-ecological landscape. Approximately 1,380 households reside within the buffer zone, relying mainly on subsistence farming and livestock rearing. These communities face significant challenges, including extreme poverty, limited job opportunities, and restricted access to potable water and food resources. Additionally, infrastructure is scarce, and healthcare services within the park and surrounding areas are insufficient, exacerbating health risks associated with untreated water sources and zoonotic diseases. Due to the absence of fences, LNP communities — primarily located in the 1,720 km^2^ buffer zone — experience frequent interactions with wildlife. The region harbors a diverse wildlife assemblage, including large herbivores such as African buffalo and kudu, as well as scavengers and predators such as vultures, hyenas, and lions [22]. Livestock play a fundamental role in the livelihoods of these communities, serving as an economic resource and a cultural asset. Ownership is typically family-based, and livestock management follows guided circuits through open grazing areas without fences, sharing the territory with wildlife [35]. During the night, livestock are kept in enclosures to protect them from predators, while during the day, they are herded to grazing areas and water sources. Constant supervision is essential to prevent losses and reduce the risk of predation. This continuous supervision, observing livestock while enclosed, and the intergenerational transmission of knowledge form a key body of knowledge for this research –particularly practical diagnostic skills, spatial memory of high-risk areas, and local disease management strategies.

This study focused on three communities located in the Pafuri area in the northern region of LNP (Fig 1), referred to here as Community 1, Community 2, and Community 3 to protect the privacy and anonymity of participants, where livestock populations are nearly double the size of the human population (Table 1). These communities are situated along the margins of the Limpopo River floodplain. This ecological corridor supports local livelihoods and facilitates wildlife movement between LNP and the northern region of KNP. This hydrological connection enhances the potential for anthrax spore dispersal through alluvial processes. These communities were selected due to several key factors that heighten their significance for studying anthrax transmission and mitigation strategies. The high livestock density and continuous interaction with wildlife, including both human-wildlife and livestock-wildlife interactions, significantly increase the risk of zoonotic disease transmission.

**Table 1:** Human and livestock population characteristics of the three study communities. Area is reported in km² and was estimated as the Minimum Convex Polygon (MCP) for each community. Human population density (people/km²) and livestock density (animals/km²) were calculated using this area and information provided by the LNP administration. Community names have been withheld in compliance with human subjects protection guidelines.

Community name	Families	Population	Livestock	Area (km²)	Population density (people/km²)	Livestock density (animals/km²)
Community 1	61	227	500	10.19	22.26	49.04
Community 2	86	350	600	11.55	30.29	51.91
Community 3	170	620	1100	28.76	21.55	38.23

**Fig 1:**
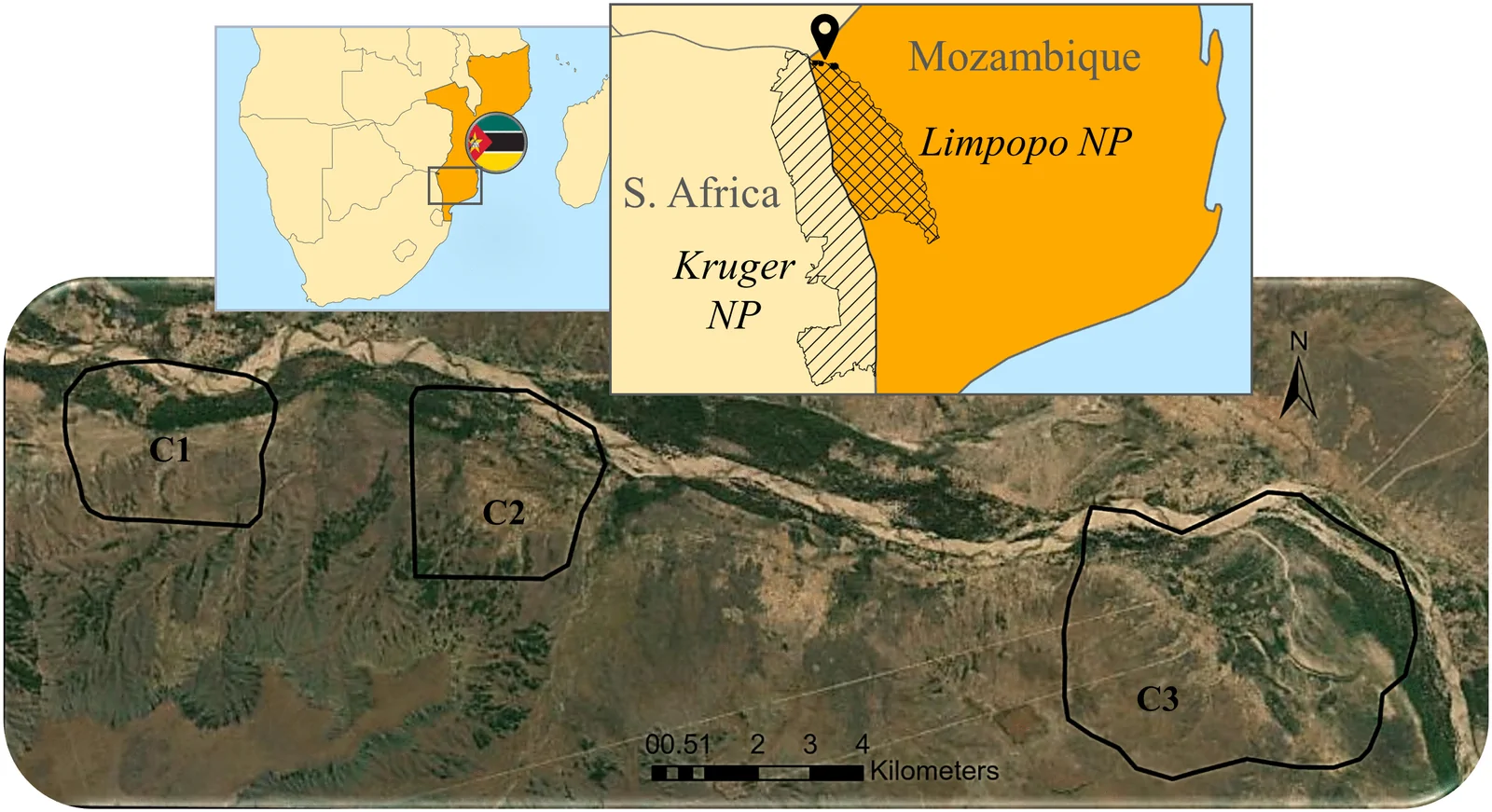
Study area located in the southwestern part of Mozambique. For the purposes of this research, the three communities (black borders), left to right, are labeled: Community 1, Community 2, and Community 3. Basemap source: Esri, Vantor, Earthstar Geographics, and the GIS User Community (https://www.arcgis.com/home/item.html?id=10df2279f9684e4a9f6a7f08febac2a9#overview). Country Boundaries were acquired from United Nations World Administrative Boundaries under Open Government License v3.0. (https://public.opendatasoft.com/explore/dataset/world-administrative-boundaries/export/?flg=en-us). Community boundaries shown in black are study-specific Minimum Convex Polygons (MCPs) generated by the authors, as described in the Methods.

Additionally, the ecological characteristics shared with northern KNP, an area known for recurrent anthrax outbreaks, make this region particularly suitable for the persistence and spread of *B. anthracis*. The proximity to northern KNP, where numerous anthrax outbreaks have been documented, further reinforces the relevance of this study area. These factors make the Pafuri area a crucial site for analyzing transmission dynamics and developing effective mitigation strategies.

### 2.3 Data collection

#### 2.3.1 Ecological suitability.

Previous studies [8,17,20] developed ecological suitability maps for anthrax occurrence in African savannas based on soil properties (pH, calcium levels), climate (temperature and precipitation), vegetation (Normalized Difference Vegetation Index [NDVI] or Enhanced Vegetation Index [EVI]), distance from water bodies (permanent and seasonal), and topography. The location and size of our study area impose two major limitations on replicating previously used methods: the absence of field data on anthrax cases and the lack of high spatial resolution data for calculating ecological predictors that reflect the size of the selected communities—issues largely due to limited monitoring systems in these areas.

The lack of ground-truth data can be addressed by using global satellite and model-based datasets to estimate ecological drivers, which are essential data sources for remote areas [24,36]. In this study, we selected 27 environmental variables based on their importance for the survival and distribution of anthrax spores. Climate variables have been identified to play a major role in the spread of the disease [8,17], and here, the highest spatial resolution global climate dataset, WorldClim [37], was used to test 19 climate variables based on temperature and precipitation averaged over various seasons. In the absence of soil sampling, we utilized the SoilGrids product [38] to retrieve five chemical (organic carbon, total nitrogen, soil pH, cation exchange capacity) and physical (soil texture: clay) properties. As anthrax-positive cases are usually located at low altitudes, elevation was calculated using the Shuttle Radar Topography Mission (SRTM) 1 Arc-Second Global dataset at 30 m resolution accessed from USGS EarthExplorer. Increased transmission has been associated with proximity to water sources, which we calculated as Euclidean distance from rivers utilizing the HydroRIVERS dataset [39]. Mean NDVI as a proxy for vegetation density was estimated using all available Sentinel-2 images at 20 m resolution from 2017 to 2023 within Google Earth Engine.

#### 2.3.2 Participatory mapping.

Participatory mapping was carried out in June 2024. During the fieldwork, the research team—composed of members from diverse cultural backgrounds, including English and Portuguese speakers—trained local data enumerators and facilitated pre-mapping sessions to express gratitude and gather community feedback. Community-based data enumerators facilitated the participatory mapping activities. These enumerators were selected to minimize cultural distance and strengthen local capacity-building [40]. Each enumerator received daily training to enhance their skills and actively contribute to the research. Participatory mapping sessions were conducted in four simultaneous groups per community, each representing a livestock-owning family, and were intentionally diverse in age and gender. There were three participatory mapping sessions (1 per community) involving four families per community (twelve families in total). Large printed maps (90 x 100 cm) were placed on spacious tables for group use, with participants standing around them, while chairs were provided for elderly members when needed. A moderator facilitated the sessions in Portuguese, with data enumerators translating into Shangana. Groups marked their responses directly on the maps using specific symbols linked to each question (S1 Material), ensuring clarity and traceability. Before starting the activity and trying to improve the cartographic literacy of participants [41], the maps were introduced through an orientation activity. Participants identified key spatial landmarks such as the Limpopo River, roads, the local school, and the activity venue. They were encouraged to rotate the maps, locate cardinal points, and view them from different perspectives to enhance spatial awareness (Fig 2).

**Fig 2:**
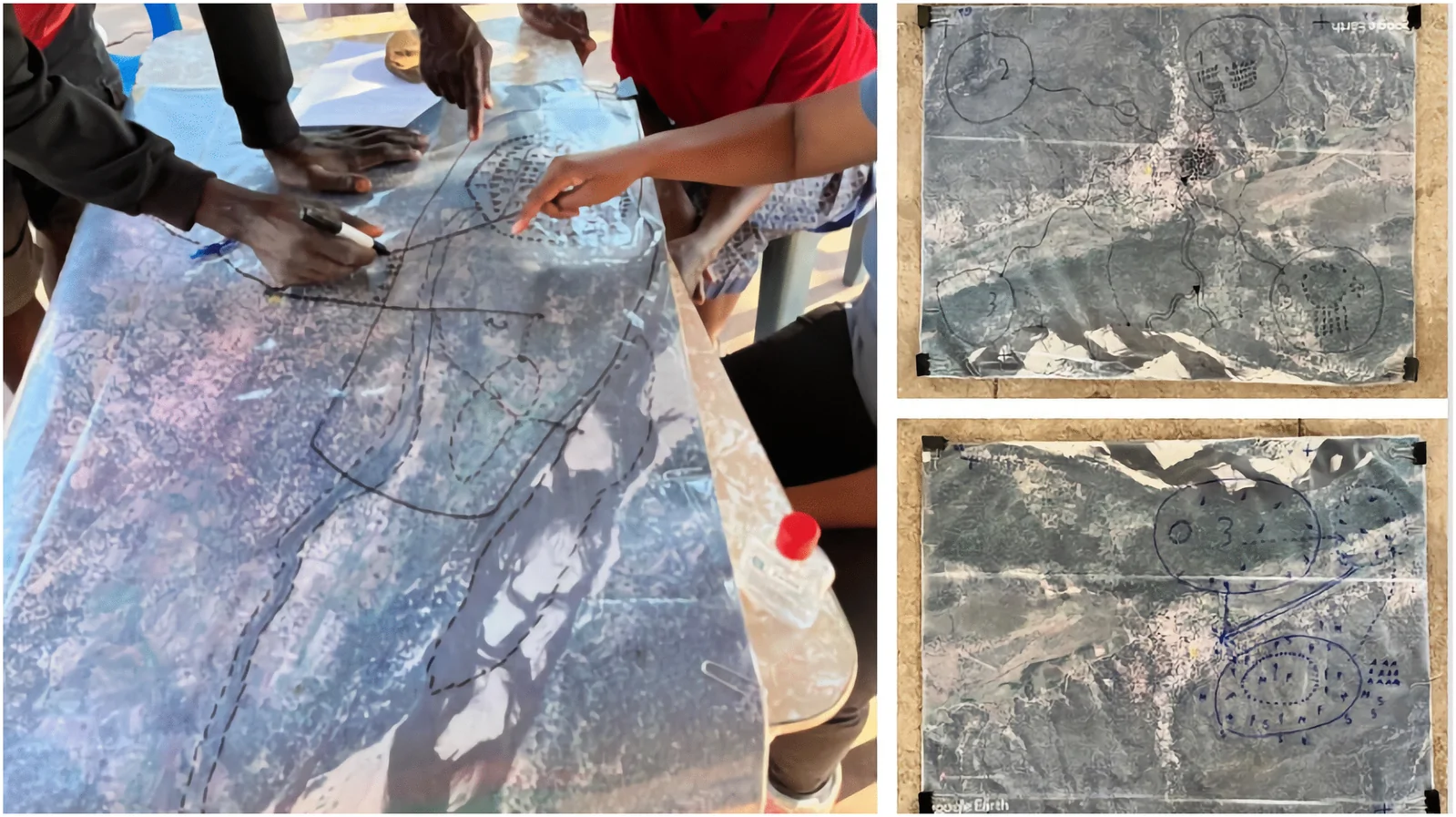
Participatory mapping activities in the communities. Left: community members marking the various areas asked about during the activity. Right (above and below): examples of maps generated by two different groups in the same community. Photographs by the authors.

Participants’ markings and drawings were made on transparent sheets placed over the maps using permanent markers. Each sheet was secured with clips to prevent movement and aligned with specific reference points on the base maps to ensure spatial accuracy and avoid displacement. During the activity, corrections were made as needed using alcohol-based erasers; however, once the activity was completed, the markings remained permanent and were not erased. The mappings resulted in 12 plastics (1 per family) with spatial information. The central part of the mapping involved asking community members to provide spatial information regarding their livestock practices, interactions with wildlife, and the presence of diseases. These activities aimed to identify areas where zoonotic disease transmission events, particularly anthrax, could occur due to livestock presence in grazing, hydration, transit zones, or through interactions with wildlife. The mapping questions considered the features presented in Table 2. After completing the mapping activity, participants shared their group’s map with the other participants.

**Table 2:** Risk layers used in the spatial analysis of anthrax transmission, ordered by assigned risk value (A = high, B = medium, C = low). Each layer corresponds to a mapped spatial feature during participatory mapping sessions and represents a potential ecological or behavioral factor contributing to the exposure and transmission of anthrax among livestock, whether through ecological sources or interactions with wildlife.

Risk type and value	Layer	Description
A [10]	Suspected anthrax zones for livestock	Areas where anthrax is alleged to have affected livestock
	Suspected anthrax zones for wildlife	Areas where anthrax is alleged to have affected wildlife
A [7]	Areas where livestock get sick	Areas where livestock have shown symptoms of any disease
	Locations of natural wildlife deaths	Observed sites where wild animals have died
B [5]	Grazing areas	Open areas where livestock feed during the day
	Shared spaces with wildlife	Areas where livestock and wildlife are known to co-occur
	Water sources for livestock	Permanent or seasonal water bodies used by livestock
C [2]	Livestock grazing routes	Regular paths followed by livestock to reach grazing areas
	Livestock pens	Areas where livestock are confined during the night in wooden-fenced pens
	Routes back to pens	Return routes to pens
	Water access routes	Paths used by livestock to reach water sources

### 2.4 Data analysis

#### 2.4.1 Ecological suitability.

Due to the absence of data containing the location of confirmed positive anthrax cases within the communities and surrounding areas, established methods for identifying ecologically suitable areas (e.g., Maxent) were applied using data from the neighboring park. Specifically, the assumption was that ecological threshold values calculated from the KNP anthrax database can be used for the three communities in the neighboring park in Mozambique. This assumption was based on proximity to the highly anthrax-prone region in Pafuri KNP and similar soil types.

A species distribution model, Maxent, was used to map the ecological suitability of anthrax based on the locations of confirmed positive anthrax cases in Pafuri KNP from 1992 to 2021. Prior to model fitting, the presence of collinearity among the independent variables was first assessed by calculating the variance inflation factor (VIF) using VIF > 3 as an indicator of a high correlation similar to other works [24,36]. As expected, significant multicollinearity was detected between climate variables. Uncorrelated variables were directly included in the Maxent model, whereas climate variables required additional exploratory analysis using bivariate generalized additive models (GAMs) [42]. GAMs were used to investigate the relationship between each of the remaining highly correlated variables and the presence of anthrax, following the approach of Zubkova et al. [43] in which the independent variable with the highest explained deviance was selected in the model (S2 Material).

Species distribution models were developed using the “Maxent” package in R software [44]. Following Steenkamp et al. [8], each pixel was classified according to whether it contained at least one confirmed anthrax case, regardless of the number of cases within the pixel, to minimize model overfitting. Although the resolution of the datasets from which environmental variables were calculated varied substantially, from 20 m to 1 km, the final output had a 250 m resolution, matching the SoilGrids product, to provide sufficient spatial detail within the three communities. Therefore, all rasterized layers were resampled to 250 m. The model was based on 21,978 pixels, of which 683 contained at least one confirmed anthrax case. To mitigate data imbalance, 1,000 pixels without recorded anthrax cases were randomly selected as background pixels for each model run. The resulting dataset of 1,683 pixels was randomly divided into 75% training and 25% testing data. This partitioning was repeated across 1000 independent model runs to assess model stability and performance. For each run, a Maxent model was fitted using linear, quadratic, and product feature classes. Model fitting was performed with a convergence threshold of 1 × 10^-5^ and a maximum of 5,000 iterations. The models’ performance was evaluated using the area under the receiver operating characteristic curve (AUC). Anthrax suitability prediction for the three communities was generated by projecting the fitted Maxent models onto environmental raster layers covering the study area. To reduce uncertainty associated with individual model runs, final suitability maps were obtained by averaging predictions across the 1000 models. Final suitability surfaces were clipped to each community’s Minimum Convex Polygon (MCP) to enable intra-community comparisons.

#### 2.4.2 Participatory mapping.

The plastic sheets generated during the participatory mapping sessions were scanned and georeferenced using identifiable landmarks (e.g., road intersections, buildings) that could be independently verified. Spatial features drawn by community members were heads-up digitized to create polygon and line layers in a GIS, preserving their original location and symbology. All spatial data were projected to UTM Zone 36 South to ensure spatial consistency across datasets.

Each mapped feature—such as grazing areas, watering points, livestock pens, and reported disease locations—was categorized by the research team into one of three risk exposure levels: zone A (high risk), zone B (medium risk), and zone C (low risk) (Table 2). This classification was based on established scientific literature regarding anthrax ecology and transmission pathways in savanna ecosystems. Specifically, zone A included areas where disease occurrence was observed, such as locations of sick livestock or observed wildlife deaths—consistent with community-based epidemiology approaches [4,9]. Zone B encompassed ecological and behavioral interfaces such as shared water sources, grazing areas, and known wildlife-livestock overlap zones, all of which have been identified as critical sites for anthrax transmission [8,17]. Zone C referred to areas of limited or transient exposure potential, such as livestock pens or travel routes, where animals are less likely to encounter environmental reservoirs of *B. anthracis* [12,13].

Once the risk levels were assigned, the classified features were grouped and merged to generate composite zones A, B, and C for each community. These zones were analyzed in terms of their total area (in km^2^) and proportional extent within each community’s MCP. Each zone’s spatial distribution and concentration were evaluated to detect intra-community patterns of perceived exposure risk.

In addition, raster versions of the classified risk layers at 30 m spatial resolution were created by converting the categorical values to a normalized scale. Specifically, based on participants’ testimonies, the suspected anthrax zones for livestock and wildlife were assigned a value of 10 because they represent the attribute most directly indicative of actual anthrax presence. These areas were therefore prioritized over other high-risk (zone A) areas, which were assigned a lower value of 7. Value 5 was assigned to zone B and value 2 to zone C. To allow for more gradual changes in the values of perceived risk within the space of each community, for each 30 m (1 pixel) the value was reduced by 1. The last step was converting the 1–10 range to 0.1 to 1, which allowed compatibility with the ecological suitability surface and enabled the calculation of mean participatory risk scores per pixel for each community, providing a standardized metric for comparative analysis.

#### 2.4.3 Data integration and community comparison.

We integrated ecological suitability with participatory risk values to identify areas of cumulative anthrax transmission risk and enable systematic comparison between communities. Ecological suitability values represent the relative likelihood of *B. anthracis* spore persistence based on environmental conditions, while participatory risk values reflect community-identified livestock exposure zones derived from participatory mapping. Integration was conducted by multiplying the two rasters on a pixel-by-pixel basis, producing a composite integrated risk surface for each community. This final raster captured the spatial convergence of ecological risk and livestock exposure and retained the 0.1–1.0 scale of the input layers. We derived a set of complementary indicators from the integrated surface to characterize and compare anthrax risk across communities. High-risk areas were defined as those pixels whose integrated values reached or exceeded a threshold of 0.8, indicating the simultaneous presence of elevated ecological suitability and high perceived exposure. The extent of these areas was measured in absolute terms (km²) and as a percentage of the area of each community. In addition to this raster-based quantitative analysis, we conducted a descriptive visual assessment to explore the spatial configuration of high-risk zones (e.g., clustered, dispersed, peripheral) and to compare inter-community risk patterns qualitatively.

## 3 Results

### 3.1 Ecological suitability

Anthrax suitability was modeled using 10 environmental variables with VIF < 3 (Table 3). The model performed well on the test data with an AUC score of 0.79 ± 0.02, the average score from 1000 models. The ecological suitability analysis signaled significant variations in the ecological context for *B. anthracis* persistence across Pafuri KNP (Fig 3), whereas the vast majority of the three communities were located within moderate to high suitability regions (Fig 4). We implemented the environmental suitability thresholds of Steenkamp et al. [8] to define high (0.8-1), moderate (0.6-0.8), low (0.3-0.6) suitability and not suitable areas (0-0.3).

**Table 3:** Ten environmental variables used in the Maxent model ranked by their importance.

Rank	Variable	Unit	Resolution	Source
1	Temperature Dry Quarter	C°	1 km	WorldClim
2	Temperature Seasonality	C°	1 km	WorldClim
3	Elevation	m	30 m	SRTM
4	Cation Exchange Capacity	mmol(c)/kg	250 m	SoilGrids
5	NDVI	index	20 m	Sentinel-2
6	Distance to Rivers	m	30 m	HydroRIVERS
7	Nitrogen	cg/kg	250 m	SoilGrids
8	Clay Content	g/kg	250 m	SoilGrids
9	Soil Organic Carbon Density	kg/m^3^	250 m	SoilGrids
10	Soil pH	pH	250 m	SoilGrids

**Fig 3:**
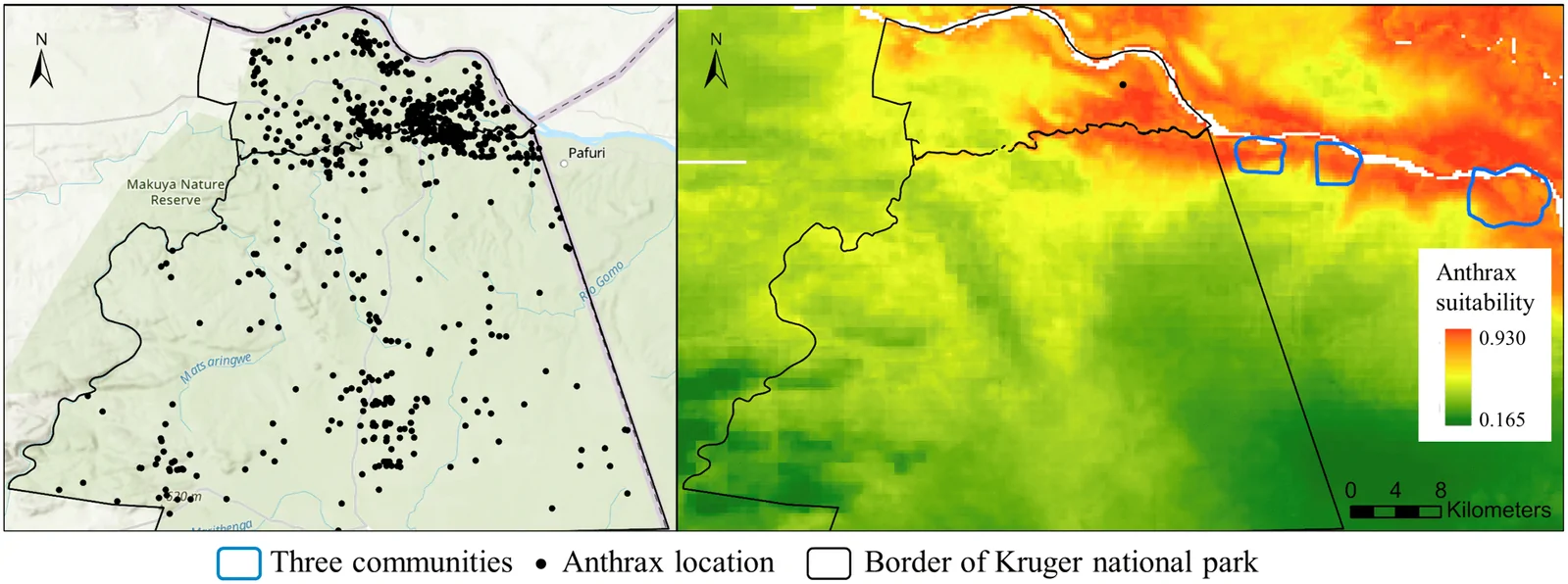
The locations of the 683 confirmed anthrax cases within Pafuri Kruger National Park (left) and the anthrax ecological suitability map for Pafuri Kruger and the three communities in Mozambique (right) based on ten environmental variables from Table 3. The locations of 7 positive cases in Pafuri from 2024 are shown on the right panel (black circle). These cases were not used for model training or testing. Basemap Esri, NASA, NGA, USGS. Sources: Esri, TomTom, Garmin, FAO, NOAA, USGS, OpenStreetMap contributors, and the GIS User Community (https://www.arcgis.com/home/item.html?id=10df2279f9684e4a9f6a7f08febac2a9#overview). Country Boundaries were acquired from United Nations World Administrative Boundaries under Open Government License v3.0 (https://public.opendatasoft.com/explore/dataset/world-administrative-boundaries/export/?flg=en-us). Community boundaries were generated by the authors as Minimum Convex Polygons (MCPs), as described in the Methods.

**Fig 4:**
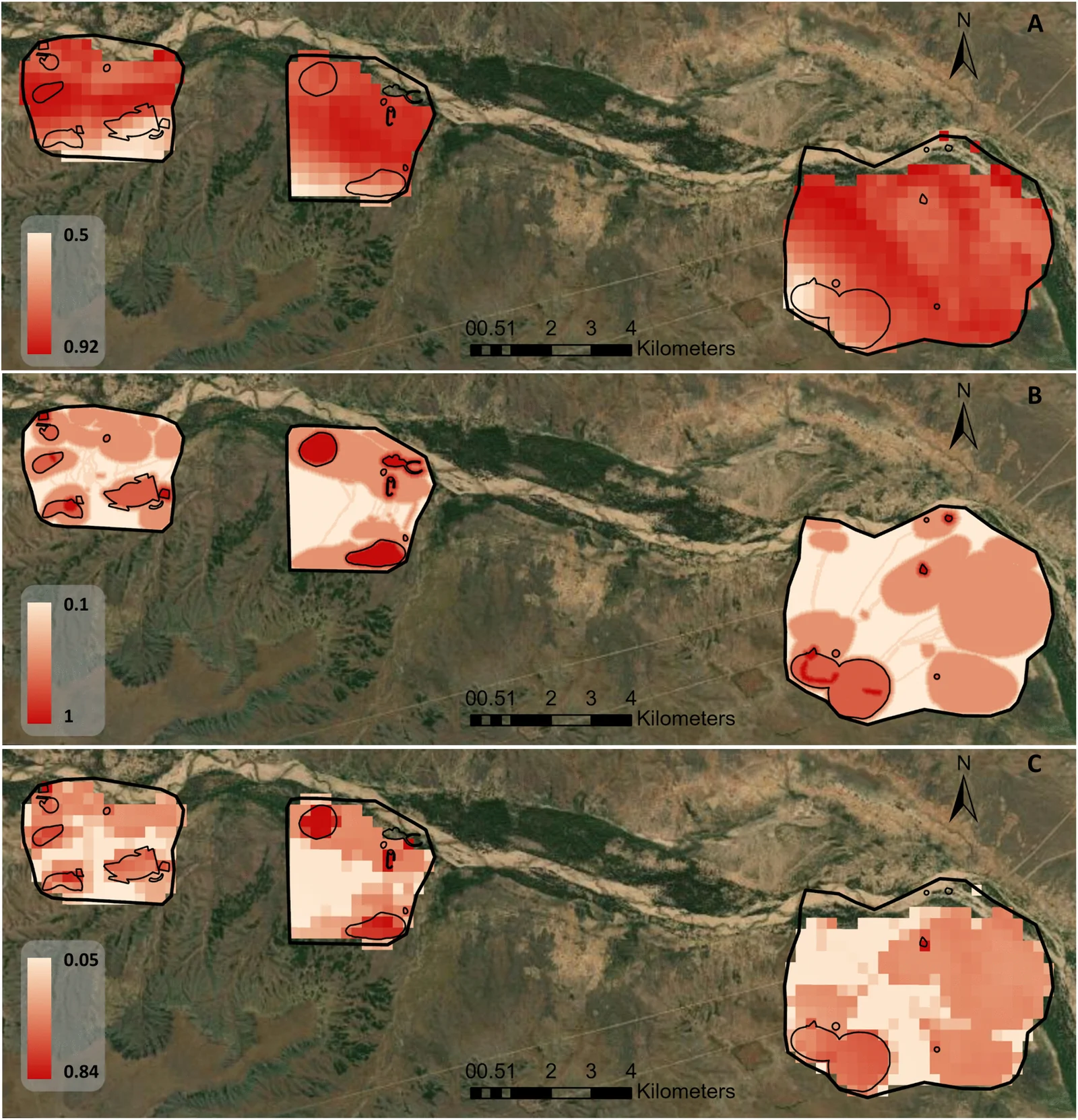
Suitability index based on 10 ecological variables (A); Perceived risk based on the results of the participatory mapping (B); Final map, which incorporates the suitability and the perceived risk maps (produced as a multiplication of both) (C). The three communities left to right: Community 1, Community 2, Community 3. Thin black borders within the communities indicate high-risk areas (zone **A)**. Basemap source: Esri, Vantor, Earthstar Geographics, and the GIS User Community (https://www.arcgis.com/home/item.html?id=10df2279f9684e4a9f6a7f08febac2a9#overview). Community boundaries were generated by the authors as Minimum Convex Polygons (MCPs), as described in the Methods.

Among the variables considered, the average temperature during the dry quarter was the primary driver of variation in suitability across the landscape, followed by temperature seasonality and elevation. It is worth acknowledging that since climate variables experienced strong multicollinearity, it can be assumed that other explanatory variables, for example, precipitation accumulated over different quartiles, can also be an important driver of anthrax presence. NDVI served as a proxy for vegetation greenness, and vegetated areas, especially closer to a water source, were considered most suitable for anthrax outbreaks. From five variables representing soil properties, cation exchange capacity showed the strongest predictive power. Specifically, within Pafuri KNP, an inverse relationship was observed between anthrax suitability and the soil’s total capacity to retain exchangeable cations such as Ca^2+^ and Mg^2+^.

Given that climatic variation across the communities is smaller than across the broader Pafuri landscape, the range in anthrax suitability can be largely explained by the elevation gradient moving south to north, which coincides with greater proximity to the river, the main water source supporting vegetation accumulation rates. However, the small scale of the communities uncovers more nuanced relationships between vegetation and anthrax suitability. The most suitable areas were concentrated closer to the middle of each community, in regions with sufficiently low elevation but with less dense vegetation than can be found near the river in the north. Areas with sparse but present vegetation were most suitable for anthrax presence. Specifically, Community 3, the spatially largest of the three, exhibited the highest overall ecological suitability with an average pixel score of 0.800, and 57.9% of its area was classified as highly suitable (Fig 4A). Community 2 followed closely, with an average suitability value of 0.793. A total of 62.5% of its territory had a pixel value ≥ 0.8. Community 1, while located the closest to KNP, had the lowest anthrax suitability, with an average pixel score of 0.768, and less than half of the area was classified as highly suitable (48.2%). Additionally, Communities 1 and 3 exhibited a more fragmented distribution of suitability values closely following NDVI, while Community 2 showed a more gradual change from north to south.

In summary, the ecological suitability analysis shows that, while all three communities exhibit favorable environmental conditions for the persistence of *B. anthracis*, no unsuitable area (pixel value ≤ 0.3) was present within any community, and significant variation in ecological suitability is evident. The highest suitability is concentrated in the central and northern sectors of Communities 1 and 2, while Community 3 is mostly highly suitable, except for a small region in the southwest. These areas are characterized by the convergence of key ecological factors, including low elevation, intermediate vegetation density, and proximity to water bodies, conditions previously associated with anthrax outbreaks in other savanna ecosystems.

### 3.2 Participatory mapping

This section presents the spatial distribution of perceived anthrax risk exposure based on participatory mapping in three communities (Fig 5). In Community 1, high-risk areas (zone A) were primarily located near the southern and western perimeter of the community. Medium-risk zones (zone B) were more extensively distributed, covering the majority of each community’s MCP. Low-risk zones (zone C) were mainly confined to linear routes used by livestock to move between grazing areas in the south and water points in the north in all three communities. In Community 2, zone A areas were concentrated in the northern and eastern sectors, displaying a spatial pattern opposite that of Community 1. The western part of the community did not contain medium-risk designations. In Community 3, the largest of the three communities, zone A represented the smallest proportion of the MCP and was concentrated in the southwestern corner. Medium-risk areas extended broadly across the central and eastern portions of the community. While still limited in overall area, zone C was more extensive in Community 3 than in the other two communities and similarly aligned with livestock transit paths.

**Fig 5:**
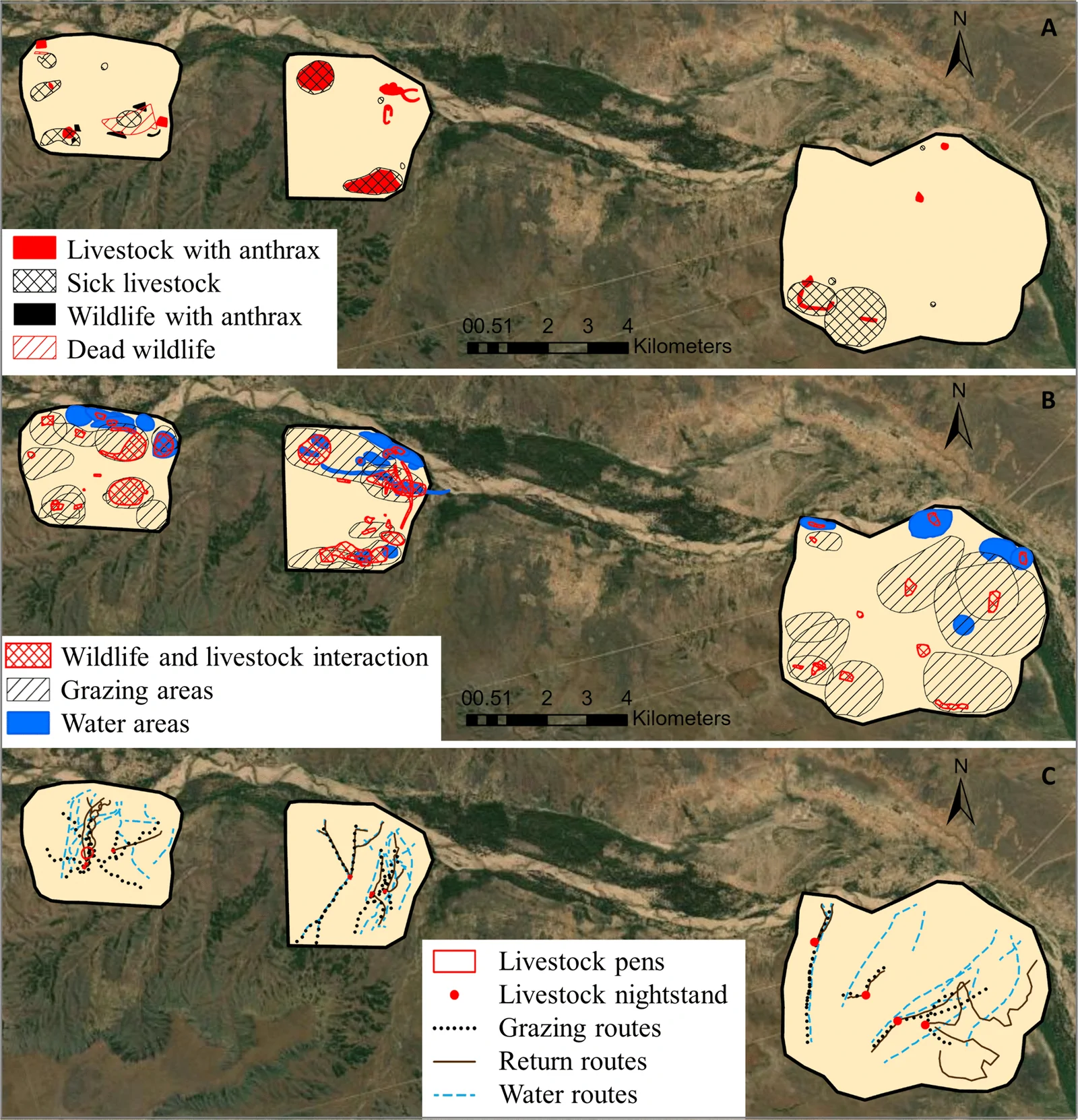
Perceived exposure risk based on the assigned risk value (A = high, B = medium, C = low) from Table 2. The top panel, the highest risk value, includes areas where livestock or wildlife have shown symptoms of anthrax or other diseases or where animals have died; the middle panel, medium risk, shows grazing and water source areas and places where livestock and wildlife interact; the bottom panel, low risk, demonstrates livestock movement. Each layer corresponds to a mapped spatial feature during participatory mapping sessions and represents a potential ecological or behavioral factor contributing to the exposure and transmission of anthrax among livestock, whether through environmental sources or interactions with wildlife. The three communities (black borders), left to right: Community 1, Community 2, Community 3. Basemap source: Esri, Vantor, Earthstar Geographics, and the GIS User Community (https://www.arcgis.com/home/item.html?id=10df2279f9684e4a9f6a7f08febac2a9#overview). Community boundaries were generated by the authors as Minimum Convex Polygons (MCPs), as described in the Methods.

In terms of surface area, zone A covered 1.81 km^2^ (16.87%) in Community 1, 2.41 km^2^ (20.95%) in Community 2, and 2.89 km^2^ (9.96%) in Community 3. While Community 3 had the largest absolute area designated as high-risk, Community 2 had the highest relative proportion of zone A within its territory. Medium-risk zones (zone B) constituted most of the mapped area in all three communities, ranging from 68.50% in Community 3 to 89.52% in Community 2. Low-risk zones (zone C) occupied the smallest areas across all communities, with proportions between 14.63% and 20.89%.

These results indicate that Community 2 was perceived as having the most significant relative transmission risk, based on the proportion of its territory identified as high-risk. In contrast, although Community 3 had a greater surface area marked as zone A, that risk was more spatially concentrated and limited in proportion to its total area. Community 1 showed an intermediate pattern, with zone A distributed along its edges and a broad presence of the medium-risk regions throughout the community.

Across the three communities, some shared spatial logics emerged in delineating transmission risk zones, while other patterns remained context specific. In all cases, zone A areas were relatively limited in spatial extent and typically concentrated in one sector of each community—along the southern and western edges in Community 1, the northeast in Community 2, and the southwest in Community 3. This suggests that, although high-risk areas were recognized across all MCPs, they were not perceived as evenly distributed or widespread. In contrast, zone B—medium-risk areas—constituted the dominant classification in all three communities, consistently occupying most of each territory and suggesting a broadly perceived baseline level of risk in areas of routine cattle activity. While comparatively minor, zone C showed a remarkably consistent association with livestock transit routes across all three sites. These similarities point to a shared understanding of spatial exposure dynamics about daily cattle movements, while the differences in the location and proportion of zone A highlight localized experiences and interpretations of disease occurrence and vulnerability.

### 3.3 Integrated risk and community-level comparison

While the ecological suitability analysis identified most of the communities as being environmentally favorable for *B. anthracis* persistence, the integrated risk model yielded a more precise set of high-risk transmission zones (Fig 4C). This reflects the combined nature of the model, which multiplies ecological suitability by community-identified livestock exposure zones. This section is organized in two subsections: A) integrated risk distribution and magnitude (focuses on the extent, concentration, and internal variation of risk accumulation) and B) associations between participatory risk and ecological suitability, which examines the spatial correspondence between participatory zone A areas and modeled ecological suitability, evaluating convergence and divergence in risk identification across the three communities.

#### 3.3.1 Integrated risk distribution and magnitude.

Overall, the areas that the ecological suitability analysis defined as highly suitable did not overlap with regions where animal deaths were suspected to be caused by anthrax based on participatory mapping (risk type A and value 10, see Table 2). Specifically, high-risk areas—defined as pixels with integrated values ≥0.8—could only be found in the northern part of Community 2. In terms of the average integrated risk, calculated as the sum of all pixel values within each MCP and divided by the number of pixels, Community 2 ranked the highest (0.324), followed by Community 1 and 3 (0.315 and 0.303, respectively). The north-south gradient in anthrax suitability was not confirmed by local communities, and moderate integrated risk areas (0.6-0.8) were located on both edges within Community 2, on the west side of Community 1, and only one pixel on the north had moderate suitability within Community 3.

#### 3.3.2 Associations between participatory risk and ecological suitability.

We also examined the degree of spatial overlap between the zone A areas identified through participatory mapping and the ecologically suitable zones modeled in the GIS analysis. Overlay analysis revealed weak spatial convergence in all three communities. In Community 1 and Community 2, 25.1% and 19.3% of the zone A area overlapped with high ecological suitability (≥0.8). In Community 3, the correspondence was the weakest, with only 7.7% of zone A intersecting with highly suitable ecological regions. Livestock disease sites identified by participants in Community 3 fell outside the modeled high suitability zones, particularly in the southwestern MCP sector. On the other hand, it is important to note that the environmental conditions in the vast majority of communities were identified as moderately suitable for anthrax, and zones A were located within those moderately suitable regions.

## 4 Discussion

By combining ecological suitability data with participatory mapping of livestock management practices, we generated a composite risk landscape that captures the environmental potential for *B. anthracis* persistence and reflects localized exposure pathways identified by community members. By linking ecological suitability (spore persistence potential) with livestock dynamics (management practices and exposure to risk landscapes), we offer an integrative workflow for anthrax risk assessment, implemented through pixel-level raster integration (Section 2.4.3) and expressed as community-specific risk surfaces (Fig 4C) and inter-community comparisons (Section 3.3). This integration responds directly to long-standing calls in the literature to incorporate context-specific knowledge into zoonotic disease monitoring and prevention strategies, especially in data-poor settings [9,17,28]. This is particularly relevant in governance contexts where the exclusion of local knowledge has historically weakened the effectiveness and legitimacy of environmental interventions [33].

Steenkamp et al. [8] reported that soil type, NDVI, and precipitation contributed most to anthrax occurrence in KNP, particularly in its northern region from which our occurrence data were drawn for model training and projection. Similarly, Pittiglio et al. [25] identified alkaline soils, NDVI, and precipitation, in addition to livestock density, as the main predictors of anthrax suitability in West Africa. Host abundance or density was further emphasized by Deka et al. [27], who modeled the global ecological niche of anthrax, and by Arede et al. [26], who showed that incorporating ruminant population data with environmental variables improved model predictions of anthrax suitability in the Black Sea Basin. Building on this evidence, our study integrated participatory mapping with environmental variables to refine anthrax risk modeling. In Communities 1 and 2, ecological suitability for *B. anthracis* was largely structured by the main environmental gradients captured in the Maxent model (temperature during the dry quarter, temperature seasonality, elevation, NDVI, distance to rivers, and soil proxies), while participatory mapping added an exposure layer that helped contextualize and spatially qualify these patterns. In Community 3, suitability was high overall but more internally heterogeneous, largely associated with vegetation gradients, further underscoring the value of complementing landscape-scale suitability with locally grounded information on livestock use and exposure pathways.

*Bacillus anthracis* is a soil-borne pathogen whose endospores enable long-term environmental survival [2]. Ecological suitability studies in Zimbabwe [45], KNP [8], Ghana [46], Kazakhstan [47], Tanzania [1], and West Africa [25] consistently identified soil characteristics—such as soil type, calcium content, alkalinity, and pH—as well as NDVI (except Tanzania) as key environmental variables influencing *B. anthracis* persistence. In our model, however, climate and topography-related predictors captured most of the explained variation at this spatial scale, with soil properties contributing as secondary but interpretable correlates through global soil proxies (e.g., pH and related edaphic parameters). In KNP, for example, Steenkamp et al. [8] reported higher suitability in soil contexts typically associated with alkaline and calcium-rich conditions, including eutric leptosols within their soil classification framework. Leptosols are shallow soils limited in depth by continuous hard rock or highly calcareous material (CaCO_3_ > 40%). Nath and Dere [48] similarly reported that soil type and texture, combined with geochemical conditions, delineate areas most suitable for *B. anthracis*. In Tanzania, Mwakapeje et al. [1] identified fluvisols, lithosols and solonetz soils, underscoring how alluvial and hydrologically structured soil settings can shape suitability patterns. Together, these studies highlight how soil geochemistry and vegetation structure interact with broader landscape gradients—an interaction consistent with our finding that suitability concentrated in low-lying, river-proximate areas with intermediate vegetation density—thereby reinforcing the relevance of geochemical factors for mapping and managing anthrax risk across diverse landscapes.

Vegetation quantity in savannas increases linearly with rainfall [49], and a similar relationship exists between NDVI and vegetation productivity [50], reflecting the strong association between NDVI and rainfall in African savannas [51]. NDVI has been widely used to predict the distribution and abundance of large herbivores. As a measure of “greenness,” NDVI serves as a proxy for grazing and browsing resources and has therefore been widely used to infer where large herbivores are likely to concentrate across space and time [52,53]. Because anthrax transmission occurs mainly through ingestion of spores while feeding, herbivore aggregation around forage patches increases exposure risk. Indeed, anthrax outbreaks in both West Africa and KNP have been associated with post-dry-season vegetation flushes, when herbivores crowd onto limited forage patches [8,25]. In our study area, ecological suitability is expressed as a gradient where higher values concentrate in low-lying, river-influenced zones with accessible forage conditions, rather than being restricted to a small set of discrete hotspots. This supports the view that surveillance strategies informed by ecological suitability should consider broader zones of concern in transboundary conservation landscapes characterized by high wildlife mobility and frequent livestock–wildlife interactions [5,20].

Ecological suitability analysis indicated that Communities 1, 2, and 3 are all located within landscapes broadly favorable to *B. anthracis* persistence. Communities 1 and 2 showed more spatially concentrated high-suitability patches, whereas Community 3 exhibited high overall suitability with greater internal heterogeneity linked to vegetation gradients. Participatory mapping, in turn, identified exposure zones shaped by cattle keeping practices, grazing and water use, mobility routes, and locally recognized disease events. This complements evidence that incorporating host abundance or livestock density can improve anthrax suitability models [25–27], by highlighting how livestock management and everyday movements structure potential contact with environmental reservoirs and wildlife interfaces. Across the three communities, exposure categories played distinct roles in shaping the integrated risk surface: high-exposure areas provided the most localized signals of concern (often anchored in reported livestock disease events), medium-exposure areas captured routine grazing and water use across most of each territory, and low-exposure areas traced livestock transit routes that were more linear and peripheral yet critical for linking daily herd mobility with potential points of pathogen contact. Taken together, these patterns show how local knowledge helps map differentiated exposure and clarify how disease events, resource use, and mobility interact to produce spatially distinct but comparable risk configurations across communities. More broadly, participatory mapping has been used in other contexts to mobilize community observations for place-based planning, including climate-related adaptation processes [54]. In this study, participatory mapping grounded in local knowledge provides a robust, context-specific approach to revealing spatial exposure dynamics that ecological suitability modeling alone cannot capture.

Integrating ecological suitability with participatory exposure layers refined the assessment by moving from a broadly suitable landscape to a more selective composite risk surface. Under the ≥ 0.8 threshold, very high integrated risk was identified only in the northern sector of Community 2, while the remaining areas across communities were predominantly low to moderate, indicating that suitability alone does not translate into localized transmission concern. Overall, the maps showed the clearest alignment in the moderate suitability range; by contrast, the highest-suitability class did not systematically align with mapped high-exposure features, indicating that fine-scale risk emerges from how routine mobility and resource use structure contact within broadly suitable environments. Rather than a limitation or contradiction, this apparent misalignment reveals the complementary value of the integrated approach. Ecological suitability modeling captures landscape-scale environmental potential for *B. anthracis* persistence—the regional conditions that favor spore survival in soil and vegetation. This broad-scale assessment is essential for understanding where transmission could occur if livestock were to encounter the pathogen. Participatory mapping, by contrast, reveals where transmission actually occurs or is perceived to occur within those broadly suitable areas, by documenting the fine-scale livestock movements, water use patterns, and disease events that structure real exposure risk. The lack of perfect spatial alignment thus indicates that high ecological suitability alone is not sufficient to determine transmission risk; local practices and experiences further modulate exposure within environmentally favorable areas. This is consistent with evidence from other zoonotic systems showing that ecological niche models alone often over-predict risk at local scales without incorporating behavioral, social, or management dimensions [32,34]. By integrating both layers, we move beyond asking “where could the pathogen persist?” (ecological suitability alone) to identify “where may livestock be more likely to encounter it?” (participatory mapping).

The COVID-19 pandemic demonstrated the critical role of modeling in guiding public health decision-making at scale, but also underscored the need for better models, capable of integrating diverse data sources and scales [55]. In the anthrax literature, such integration has been used to support targeted intervention planning. For example, Kracalik et al. [46] applied spatial modeling in Ghana to prioritize hotspots for focused vaccination. In Mozambique, comparable strategies such as awareness campaigns, strengthened community-based surveillance, and, where feasible, targeted livestock vaccination could enhance anthrax prevention and control while reinforcing animal and public health systems in resource-limited settings.

Despite its contributions, this study presents limitations that must be acknowledged. First, the spatial accuracy of participatory mapping is inherently constrained. Although georeferencing and orientation exercises were implemented to enhance precision, community-drawn features remain subject to generalization, scale distortion, and interpretive variation. Spatial imprecision is a recognized feature of community-based health mapping and should be considered when evaluating proximity relationships or estimating fine-scale exposure risk [56]. Second, although standardized, the ecological suitability component relied on global datasets that might be too coarse for our study area and not tailored to localized risk modeling. The absence of fine-scale environmental data— such as site-specific soil calcium content or in situ pH— necessitated reliance on global edaphic proxies and indirect inference about local geochemical conditions, limiting the precision of the resulting maps. Third, and perhaps most critically, the region lacks robust verified records of anthrax outbreaks or laboratory-confirmed cases. Recent anti-poaching security protocols and delayed laboratory feedback have limited the availability of reliable GPS coordinates for carcass sampling in KNP, while sampling effort remains spatially uneven. Although confirmed anthrax-positive records are not available for the three communities, a small set of seven confirmed cases from Pafuri KNP in 2024 could be mapped at the scale of our analysis and fell within a highly suitable area of our ecological suitability surface (Fig 3; raster value = 0.82). While this cannot substitute for model validation, it provides a limited external consistency check and modestly increases confidence in the study’s outputs.

Notably, this last limitation underscores one of the study’s central contributions. Integrating ecological suitability modeling with participatory mapping offers a practical proxy for anticipating zoonotic risk in settings where outbreak data are absent, incomplete, or unreliable. By combining landscape-level environmental potential for *B. anthracis* persistence with locally grounded knowledge of cattle management, mobility, and disease experience, the approach adds socially meaningful spatial granularity and supports more context-specific prioritization for surveillance and prevention. This is particularly relevant for conservation landscapes such as the northern LNP, where fragmented veterinary infrastructure, frequent livestock–wildlife interactions, and limited surveillance capacity demand spatially targeted and socially grounded interventions.

Overall, this study illustrates how combining ecological and participatory data can provide a scalable framework for anthrax risk prioritization and management in under-resourced rural areas. Beyond anthrax, this integrative framework could be applied to other environmentally persistent livestock and zoonotic diseases where surveillance data are scarce, such as brucellosis, Rift Valley fever, or foot-and-mouth disease in similar transboundary conservation landscapes across sub-Saharan Africa or Central Asia. The core requirements—environmental covariates from global datasets and participatory mapping capacity—are widely available, making the approach transferable to resource-limited settings beyond southern Africa.

## 5 Conclusions

This study demonstrates the utility of integrating ecological modeling and Indigenous knowledge through GIS to identify spatial configurations associated with anthrax transmission risk in northern LNP. By combining ecological suitability data with participatory mapping of livestock practices and abundance, we produced community-specific risk surfaces in a region with limited outbreak records and constrained veterinary infrastructure. This integrative approach can be readily adapted to other under-surveilled, high-risk regions where zoonotic threats intersect with limited infrastructure and strong local ecological knowledge.

Although ecological suitability was broadly favorable across the study area, the integrated risk surface refined this pattern by prioritizing a smaller set of locations where environmental conditions intersected with locally identified exposure dynamics. Overall spatial correspondence between modeled high suitability and perceived high-exposure zones was limited, highlighting that environmental potential and lived exposure do not necessarily co-locate at fine scales. This finding underscores the complementarity of ecological proxies and participatory knowledge for identifying locally meaningful exposure pathways within broadly suitable landscapes. Together, these findings support the value of participatory inputs not as substitutes for ecological modeling, but as essential components for fine-scale prioritization of surveillance and prevention in data-scarce settings.

The integrated approach supports three key prevention strategies: risk communication tailored to locally identified exposure zones, targeted livestock vaccination campaigns, and participatory community-based surveillance systems. For Indigenous communities living in high-suitability areas, these risk maps provide a tool for awareness and early action, supporting livelihoods that depend on healthy livestock. As climate change and land-use pressures intensify, approaches that combine scientific modeling with local knowledge will be increasingly essential for safeguarding human and non-human health in complex socio-ecological systems.

## Supporting information

S1 Material Participatory risk mapping protocol and consent form. (PDF)

S2 Material List of all 27 environmental variables considered for the ecological suitability modeling, including source and deviance explained by each climate variable in the GAM analysis. (PDF)
