# Supplementary material for: Integrating indigenous knowledge and ecological modeling to understand anthrax transmission risk
Source: PLoS Negl Trop Dis. 2026 Nov 10;20(11):e0014796. doi: 10.1371/journal.pntd.0014796 (PMC13750556; doi:10.1371/journal.pntd.0014796)
Supplement: Supplementary file 1 [file pntd.0014796.s001.pdf]

SUPPLEMENTARY MATERIAL 1

Participatory Risk Mapping of Livestock Grazing in Endemic Anthrax Zones

General description and goal

The objective of this activity is to use participatory risk mapping to advance holistic understanding about the socio-environmental characteristics impacting disease - risk transmission, with emphasis on anthrax in Mozambique.

Considering that livestock belong to families, we are interested in identifying specifically the areas of families that play a strong role in livestock management in each community.

Each family must perform the activity on a separate map, so that the work will be done with 4 maps at the same time.

Study sites
Mozambique: Communities [anonymized] (Pafuri area)

Participants

Recruitment

Participants will be recruited by the designated data enumerator using word of mouth approximately 1-2 months before data collection. The designated data enumerator will discuss the participatory mapping activity with community leaders and ask for the 4 families in each community that have the most livestock or are recognized in the community as such. Each of the 4 families will designate persons they consider to have the most experience in livestock management within their own family. We will work with the 4 families at the same time. Each family group should have gender and age parity.

The men and women should be current community residents and over 18. There are no literacy requirements, but they should have knowledge about livestock grazing.

The data enumerator will review and confirm the desired language of the participatory mapping. UMD researcher will review the type of space desired for the activity, ideally a place with tables or a floor where paper maps can be drawn without strong wind. UMD researcher will review and confirm the dates of the visit.

Procedures

The same procedure will be used in all communities.

1. Participants will convene and be asked for verbal consent; they will be given a copy for their records if they like.
2. As a large group, participants will be briefed on the activities for the day.
3. Participants will be assigned to one of 4 paper map groups.
4. Participants will conduct the participatory risk mapping.

5. Researchers will record the number of women and men on each map and the estimated age of participants.
6. Participants will be asked to share highlights from their experience as a large group if they would like to.
7. Researchers will collect the paper maps.
8. Maps will be digitized.
9. Data will be analyzed.
10. Original paper maps will be returned to communities within a maximum of 12 months.

Supplies

1. Base maps with simple satellite information that is understandable to participants.
2. Transparent plastic will be placed over the base map. The participants will draw on this plastic having base maps as references. These plastics will have some spatial reference marks (previously marked by the researchers) that allow for maintaining the spatial connection with the base maps that will be underneath.
3. Markers
4. Sticker dots
5. Wide masking tape
6. Post it notes

Institutional Review Board
1204 Marie Mount Hall ● 7814 Regents Drive ● College Park, MD 20742 ● 301-405-4212 ● irb@umd.edu

Consent to Participate Participatory Mapping

**Project Title**	*ISN2: Supplement. Reducing security risks from the intersection of wildlife trafficking and biosafety from zoonotic pathogens in Mozambique's Limpopo National Park*
**Purpose of the Study**	Dr. Meredith Gore is conducting this research at the University of Maryland, College Park, USA. We are inviting you to participate in this research project because you live close to the Greater Limpopo Transfrontier Conservation Area. This research project aims to learn more about the connection between livestock, wildlife, and human health.
**Procedures**	The research involves participatory mapping, where you will be asked to map your knowledge about the places where livestock graze based on your lived experience and expertise. This will take about 3 hours and will be conducted here in the [community center.] The knowledge will be collected on paper maps. The researchers will ask questions about where livestock graze, where they drink water, where they move and how they interact with wildlife. After the activity, participants will be able to share their experience with the rest of the groups. Some participants may be invited to participate in a member checking conversation in about one year. Researchers may take observational notes.
**Potential Risks and Discomforts**	There are no foreseeable risks from participating in this research study.
**Potential Benefits**	There are no foreseeable direct benefits from participating in this research. We hope that, in the future, other people from this community or another might benefit from this study through improved understanding of local perceptions about the transmission of diseases between the environment, wildlife and livestock.
**Confidentiality**	Your identity will be known to other participants and the researcher(s) cannot guarantee that others in the group will respect the confidentiality of the group. No personally identifying information will be collected during the mapping activities.
**Right to Withdraw and Questions**	Your participation is completely voluntary. You may choose not to take part at all. You may skip any question that you like. If you decide to participate in this research, you may stop participating at any time. You will also be able to enter and exit the activity as many times as you wish. If you decide not to participate in this study or if you stop participating at any time, you will not be penalized or lose any benefits to which you otherwise qualify. If you have questions, concerns, or complaints, or if you need to

	report an injury related to the research, please contact the investigator: Meredith Gore Department of Geographical Sciences, 4600 River Road, Rm. 322, Riverdale, MD 20742, USA; gorem@umd.edu; +1 607 592-0422.	
**Participant Rights**	If you have questions about your rights as a research participant or wish to report a research-related injury, please contact:<br><br>University of Maryland College Park, USA<br>Institutional Review Board Office<br>1204 Marie Mount Hall<br>College Park, Maryland, 20742<br>E-mail: irb@umd.edu<br>Telephone: 301-405-0678<br>IRB: 2068333-1<br>For more information regarding participant rights, please visit:<br>https://research.umd.edu/research-resources/research-compliance/institutional-review-board-irb/research-participants.<br>This research has been reviewed according to the University of Maryland, College Park IRB procedures for research involving human subjects.	
**Statement of Consent**	Are you ready to decide whether or not to participate? By verbally agreeing to take part in this study, you acknowledge you understand and accept all of the information provided, including that you are at least 18 years of age. Do you agree to participate in the study? Please state yes or no. Here is a blank copy of the consent form for your records.	
**Verbal Consent and Date**	**VERBAL CONFIRMATION (YES/NO)**	

Participatory Mapping Script

Welcome to this participatory mapping activity! This mapping activity is a fun event and happy space for all families to share their lived experiences and expertise about livestock, space, and wildlife. We are going to be doing a family mapping activity. There are no right or wrong answers! We are going to work with the same map but from two different seasons: rainy and dry. So two groups are going to work with the same map that is of the rainy season and the other two groups with the same map that is of the dry season. The idea is to do the same activity among the different families. Then, the families that want to can share the results with the other families.

First, we would like each family to sit at a different table [*point to 4 tables with identical sets of 4 paper maps, stickers, markers and post it notes, which should be set up in advance*]. We would like to ask you to stay with your family at the table for the entire time. Each family must respond according to its own grazing practices, that is, without considering the practices of other families

[*pause, ask if there is a need for clarifying questions/comments, circle room as participants map*]

Second, as a family, we would like you to review all the maps and orient yourselves according to important places to your family, such as a sacred tree, ancestorial markers, market, school, soccer field, livestock cradle, clinic, or main routes.

We are not interested in how you know where these places are—maybe you have been there yourself or maybe you have heard about it from a family member or a friend.

[*pause, ask if there is a need for clarifying questions/comments, circle room as participants map*]

Now, we would like you to draw important places for your family's livestock grazing. By grazing areas, we mean the areas where your livestock regularly go to graze in all seasons. By livestock, we include cattle, male and female.

In addition to the maps that we provide on each table, there is a guide with legends for each table. The guide will help you better understand the graphical representations we want you to make on the maps to know what is answered in each question. The data enumerators will help you with this. Except for 2 questions, the vast majority will be answered with drawings on the map.

1. **Mark the pens where the livestock spends the night**. Mark it with an asterisk.
2. **Where are the areas where your livestock graze during the day? If there are different zones, mark them all**. Mark them with a large continuous line polygon.
3. **Indicate the preference of the different zones for livestock**. Mark those that are most preferred with a 3, those with medium preference with a 2, and the least preference with a 1.
4. **Mark what makes them important**, for example, the best grass, closest to your home, or water to drink. Write it on the guide (in the place of response number 4 of "the guide")
5. **Mark the places where the livestock drink water. If you have many intake places**. Mark them with a dashed line polygon.
6. **Mark the routes used by livestock to graze**. Consider marking the routes from the pens (asterisk) and between the different grazing areas with a continuous line arrow.
7. **The same but to drink water**. Mark them with a dashed line arrow.
8. **The same goes for the return to the pens**. Mark it with a dotted arrow.
9. **Mark the areas where livestock get sick most regularly**. Polygon with small circles.
10. **Map the areas where you think livestock have become ill with anthrax**. Mark these areas with A.

11. **Please identify the locations where your livestock share space with wild animals to eat, drink, and sleep**. Use the letter F.
12. **Mark where wild animals live near the community**. Use also the letter F.
13. **Mark where wildlife regularly dies of natural causes**. Use the letter M.
14. **Mark where wild animals have been found dead from anthrax**. Use the letter S.

(Not marked on the map) Symptoms of sick livestock. Write on the back of the sheet.

Finally, we would like to do a show-and-tell if the group is interested. Please raise your hand high if you would like to participate.

□ If yes, can each group please select one map and two people to come to the front of the room and share. Then move to end.

□ If no, move to end.

Thank you very much for your participation in this mapping activity. We have learned a lot from you and hope you have found this to be an enjoyable experience. Our plan is to return here in as short a time as possible and present to you a single map with ALL of the information on it. We will use a computer to put the information together. After we use a computer to put the information together, we would like to ask your help in addressing any mistakes or new information that will help us improve our learning. We will also share this information with decision makers to help support their sustainable activities.
