# Supplementary material for: Integrating indigenous knowledge and ecological modeling to understand anthrax transmission risk
Source: PLoS Negl Trop Dis. 2026 Nov 10;20(11):e0014796. doi: 10.1371/journal.pntd.0014796 (PMC13750556; doi:10.1371/journal.pntd.0014796)
Supplement: Supplementary file 2 [file pntd.0014796.s002.pdf]

SUPPLEMENTARY MATERIAL 2

Table SM2. A list of all 27 environmental variables considered for the ecological suitability modeling. Asterisk indicates variables that were selected for the final Maxent model. All the climate variables (1-19) exhibited some degree of multicollinearity and required additional exploratory analysis using bivariate generalized additive models (GAMs). Among sets of highly correlated variables, the one that is a better predictor for anthrax presence (i.e., higher explained deviance, reported in the last column) was selected in the final model.

	Variable	Unit	Source	Deviance explained, %
1	Annual Mean Temperature	C°	WorldClim	8.66
2	Mean Diurnal Range	C°	WorldClim	3.84
3	Isothermality	C°	WorldClim	0.45
4	Temperature Seasonality*	C°	WorldClim	5.39
5	Max Temperature of Warmest Month	C°	WorldClim	7.55
6	Min Temperature of Coldest Month	C°	WorldClim	1.47
7	Temperature Annual Range	C°	WorldClim	4.2
8	Mean Temperature of Wettest Quarter	C°	WorldClim	8.32
9	Mean Temperature of Driest Quarter*	C°	WorldClim	10.3
10	Mean Temperature of Warmest Quarter	C°	WorldClim	8.32
11	Mean Temperature of Coldest Quarter	C°	WorldClim	10.3
12	Annual Precipitation	mm	WorldClim	7.05
13	Precipitation of Wettest Month	mm	WorldClim	7.28
14	Precipitation of Driest Month	mm	WorldClim	5.81
15	Precipitation Seasonality	mm	WorldClim	2.78
16	Precipitation of Wettest Quarter	mm	WorldClim	6.88
17	Precipitation of Driest Quarter	mm	WorldClim	6.05
18	Precipitation of Warmest Quarter	mm	WorldClim	6.88
19	Precipitation of Coldest Quarter	mm	WorldClim	6.4
20	Soil Organic Carbon Density*	kg/m^3	SoilGrids	0.52
21	Nitrogen*	cg/kg	SoilGrids	0.58
22	Soil pH*	-	SoilGrids	3.04
23	Cation Exchange Capacity*	mmol(c)/kg	SoilGrids	0.44
24	Clay Content*	g/kg	SoilGrids	0.01
25	Elevation*	m	SRTM	10.9
26	Distance to Rivers*	m	HydroRIVERS	0.55
27	NDVI*	-	Sentinel-2	0.75
